# Fatores de Risco para Infecção da Ferida Operatória em Pacientes Submetidos à Cirurgia Cardíaca Pediátrica

**DOI:** 10.36660/abc.20220592

**Published:** 2023-12-07

**Authors:** Anna Christina de Lima Ribeiro, Rinaldo Focaccia Siciliano, Antonio Augusto Lopes, Tania Mara Varejão Strabelli

**Affiliations:** 1 Hospital das Clínicas Faculdade de Medicina Universidade de São Paulo São Paulo SP Brasil Instituto do Coração do Hospital das Clínicas da Faculdade de Medicina da Universidade de São Paulo - Cardiologia Pediátrica e Cardiopatias Congênitas do Adulto, São Paulo , SP – Brasil; 2 Hospital das Clínicas Faculdade de Medicina Universidade de São Paulo São Paulo SP Brasil Equipe de Controle de Infecção - Instituto do Coração do Hospital das Clínicas da Faculdade de Medicina da Universidade de São Paulo , São Paulo , SP – Brasil

**Keywords:** Fatores de risco, Cardiopatias congênitas, Complicações pós-operatórias, Procedimentos cirúrgicos cardíacos, Infecção da ferida cirúrgica

## Abstract

**Fundamento:**

A infecção do sítio cirúrgico (ISC) é uma importante complicação no pós-operatório de cirurgia cardíaca pediátrica associada ao aumento da morbimortalidade.

**Objetivos:**

Identificar fatores de risco para a ISC após cirurgias cardíacas para correção de malformações congênitas.

**Métodos:**

Este estudo caso-controle incluiu 189 pacientes com um ano completo e 19 anos e 11 meses, submetidos à cirurgia cardíaca em hospital universitário terciário de cardiologia de janeiro de 2011 a dezembro de 2018. Foi realizado registro e análise de dados pré, intra e pós-operatórios. Para cada caso foram selecionados dois controles, conforme o diagnóstico da cardiopatia e cirurgia realizada em um intervalo de até 30 dias para minimizar diferenças pré e/ou intraoperatórias. Para a análise dos fatores de risco foi utilizado o modelo de regressão binária logística. Significância estatística definida como valor de p<0,05.

**Resultados:**

O estudo incluiu 66 casos e 123 controles. A incidência de ISC variou de 2% a 3,8%. Fatores de risco identificados: faixa etária de lactentes (OR 3,19, IC 95% 1,26 – 8,66, p=0,014), síndrome genética (OR 6,20, IC 95% 1,70 – 21,65, p=0,004), RACHS-1 categorias 3 e 4 (OR 8,40, IC 95% 3,30 – 21,34, p<0,001), o valor da proteína C reativa (PCR) de 48 horas pós-operatórias foi demonstrado como fator protetor para esta infecção (OR 0,85, IC 95% 0,73 – 0,98, p=0,023).

**Conclusão:**

Os fatores de risco identificados não são variáveis modificáveis. Vigilância e medidas preventivas contínuas são fundamentais para reduzir a infecção. O papel do PCR elevado no pós-operatório foi fator protetor e precisa ser melhor estudado.

## Graphical abstract

**Figura Central:**
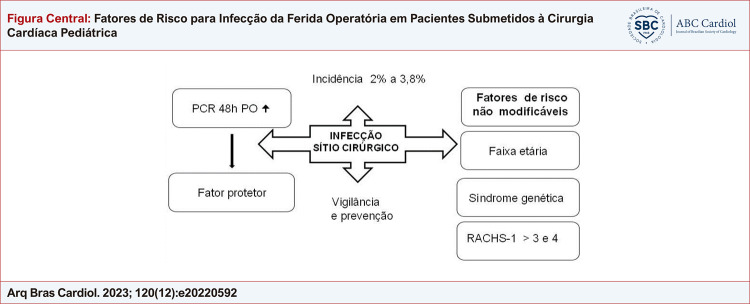
: Fatores de Risco para Infecção da Ferida Operatória em Pacientes Submetidos à Cirurgia Cardíaca Pediátrica

## Introdução

A cardiopatia congênita é considerada um relevante problema de saúde pública principalmente nos países em desenvolvimento. Apesar do aprimoramento da cirurgia cardíaca pediátrica, a demanda de serviços especializados e as limitações dos recursos humanos e financeiros são desafiadores para estes países. ^1 , 2^ A infecção do sítio cirúrgico (ISC) é uma importante complicação associada ao aumento de morbidade, aumento do consumo de antibióticos, da permanência em unidades de terapia intensiva e do tempo total de hospitalização, custos para o sistema de saúde e incremento na taxa de mortalidade. ^1 - 6^ A incidência da ISC após cirurgia cardíaca na população pediátrica, segundo dados publicados, varia de 0,2% a 4,8%. ^7^

Estudos com enfoque na identificação dos fatores de risco na população com mais de 1 ano de idade são escassos, pois o foco tem sido o período neonatal. Não há estudos com esta abordagem específica na população pediátrica no Brasil e este estudo pretende contribuir para ampliar o conhecimento sobre o assunto.

Os fatores de risco para infecção do sítio cirúrgico após cirurgia cardíaca pediátrica descritos em publicações prévias foram: idade menor que um mês, síndrome genética, escore da Sociedade Americana de Anestesiologia (ASA) alto, cardiopatia cianótica, hipotermia intraoperatória, hospitalização pré-operatória maior que 48 horas, duração da cirurgia e tempo de circulação extracorpórea (CEC), utilização de múltiplos procedimentos durante a cirurgia, número de transfusões de hemácias e manutenção do esterno aberto após término do procedimento cirúrgico. ^1 , 3 , 8^

O objetivo primário do estudo foi identificar fatores de risco para infecção do sítio cirúrgico após cirurgias cardíacas para correção de malformações congênitas com e sem circulação extracorpórea (CEC **)** em crianças maiores de 1 ano de idade e como objetivo secundário foram avaliadas a incidência e microbiologia das infecções.

## Métodos

Este estudo foi aprovado pela Comissão Científica e pela Comissão de Ética para Análise de Projetos de Pesquisa do hospital universitário terciário de cardiologia. O termo de consentimento livre e esclarecido foi dispensado.

### Pacientes

Foi utilizado o desenho de estudo caso-controle 1:2, retrospectivo para a identificação dos fatores de risco. Este estudo incluiu 189 pacientes com idade entre um ano completo e 19 anos e 11 meses, submetidos à cirurgia cardíaca realizada em centro de referência universitário especializado em assistência de alta complexidade em cirurgia cardiovascular pediátrica; no período de 01 de janeiro de 2011 a 31 de dezembro de 2018, sendo 66 casos e 123 controles. De acordo com a Organização Mundial de Saúde (OMS), a faixa etária da adolescência se situa entre 10 e 19 anos de idade. ^9^ Esta foi a padronização adotada no estudo.

### Critérios de inclusão

- Definição de caso: Paciente portador de cardiopatia congênita com idade entre um ano completo e 19 anos e 11 meses, com infecção do sítio cirúrgico após cirurgia cardíaca.
- Definição de controle: Paciente portador de cardiopatia congênita com idade entre um ano completo e 19 anos e 11 meses, submetido a cirurgia cardíaca sem infecção do sítio cirúrgico.

### Critérios de exclusão

- Pacientes neonatos (28 dias) e lactentes até o primeiro ano de vida (29 dias de idade até 11 meses e 29 dias).
- Pacientes submetidos à cirurgia cardíaca por diagnósticos diferentes de cardiopatia congênita como: cardiomiopatias, pericardiopatias, tumores cardíacos, doença reumática crônica, pacientes indicados para transplante cardíaco, pacientes indicados para colocação de dispositivo eletrônico ou de dispositivo de assistência circulatória na ausência de cardiopatia estrutural congênita.

### Seleção de casos e controles

Todos os diagnósticos de ISC foram confirmados pela equipe da Unidade de Controle de Infecção Hospitalar (UCIH) do hospital universitário terciário de cardiologia em conformidade com os critérios diagnósticos definidores segundo a ANVISA e o CDC. ^10 - 12^

Para cada caso foram selecionados dois controles e esta combinação foi baseada no diagnóstico da cardiopatia e com cirurgia realizada em um intervalo de até 30 dias para minimizar diferenças pré e/ou intraoperatórias. Os controles foram definidos por sorteio utilizando o programa Excel. Os diagnósticos das cardiopatias congênitas foram agrupados em categorias de acordo com a base patogênica, fisiopatológica e saturação arterial de oxigênio totalizando quatro categorias: grupo 1) cardiopatias congênitas acianóticas obstrutivas, grupo 2) cardiopatias congênitas acianóticas com desvio de sangue da esquerda para a direita, grupo 3) cardiopatias congênitas cianóticas com hipofluxo pulmonar e grupo 4) cardiopatias congênitas cianóticas com hiperfluxo pulmonar. ^13^ Foi realizado o registro e análise das variáveis de exposição demográficas, clínicas e laboratoriais pré, intra e pós-operatórios segundo revisão da literatura e relevância clínica e biológica ( Tabela 1 ). Considerando que a transfusão de hemoderivados pode ser um fator de risco importante, ter recebido pelo menos uma unidade de qualquer hemoderivado foi considerado como potencial fator de risco.

**Tabela 1:** – Fatores de risco para infecção do sítio cirúrgico nos pacientes com um a 19 anos de idade submetidos à cirurgia cardíaca de 2011 a 2018: análise univariada

Variável de exposição	Grupo Caso (N=66)	Grupo Controle (N=123)	Razão de chances - OR (IC 95%)	p
**Dados pré-operatórios Faixa etária, N (%)**				
Lactente (1-2 anos)	31 (47%)	36 (29,3%)	2,14 (1,15 – 3,98)	0,016
Criança (3-9 anos)	14 (21,2%)	39 (31,7%)	0,58 (0,28 - 1,17)	0,128
Adolescente (10-19 anos)	21 (31,8%)	48 (39%)	0,73 (0,39 -1,37)	0,327
Magreza, N (%)*	16 (24,3%)	25 (20%)	1,25 0,61- 2,56)	0,534
Eutrofia, N (%)**	48 (72,7%)	86 (70%)	1,10 (0,56 - 2,15)	0,772
Obesidade, N (%)***	2 (3%)	12 (9,8%)	0,29 (0,63 - 1,33)	0,111
Prematuridade, N (%)****	3 (4,5%)	3 (2,5%)	1,90 (0,37 - 9,71)	0,438
Síndrome genética, N (%)	13 (20%)	7 (5,7%)	4,03 (1,53 - 10,77)	0,005
Saturação O _2_ (%)	96 (80-98)	96 (90-98)	0,91 (0,65 - 1,25)	0,570
RACHS-1 ≥3, N (%)	43 (65%)	44 (35%)	3,35 (1,79 - 6,28)	<0,001
Procedimentos pré-operatórios, N (%)	5 (7,5%)	5 (4%)	1,83 (0,53 - 6,94)	0,311
Cirurgia prévia, N (%)	33 (50%)	43 (35%)	1,86 (1,01 - 3,41)	0,046
Total de dias internação pré-operatório, N (%)	3 (1,75-8)	3 (1-5)	1,09 (0,96 - 1,24)	0,188
**Internação pré-operatório**				
UTI, N %	5 (7,5%)	5 (4,9%)	1,70 (0,59 - 4,93)	0,324
PS, N %	7 (10%)	8 (6,5%)	1,59 (0,47 - 5,45)	0,454
Enfermaria, N (%)	57 (86%)	115 (93%)	0,44 (0,16 - 1,20)	0,110
**Laboratorial Pré-op**				
Hb (11,0 a 14,5 g/dL)	13,7 (12,6-16,0)	13,8 (12,8-15,3)	1,01 (0,91 - 1,24)	0,870
**Dados intraoperatórios**				
Tempo cirurgia (minutos)	295 (225-361)	293(230-350)	1,00 (0,90 - 1,11)	0,997
CEC (minutos)	98 (59-127)	98 (59-127,7)	0,99 (0,89 - 1,12)	0,992
Clampeamento (minutos)	65 (36-87)	63 (40-99)	0,975 (0,87 - 1,09)	0,669
Hipotermia	57 (86%)	112 (91%)	0,622 (0,24 - 1,58)	0,321
Transfusão de qualquer hemoderivado, N (%)	36 (54,5%)	65 (53%)	1,07 (0,80 - 1,95)	0,823
Transfusão de hemácias, N (%)	34 (51%)	54 (44%)	1,35 (0,74 - 2,47)	0,318
ECMO, N (%)	2 (3%)	2 (1,6%)	1,89 (0,26 - 13,73)	0,529
Tórax aberto, N (%)	3 (4,5%)	2 (1,6%)	2,88 (0,46 -17,69)	0,253
Glicose sérica mínima (mg/dL)	95 (85-110)	96 (84-109)	1,02 (0,92 - 1,14)	0,604
Glicose sérica máxima (mg/dL)	169 (131-191)	164 (131-195)	1,02 (0,92 - 1,13)	0,743
**Dados pós-operatório - laboratorial**				
**Pós–operatório imediato**				
Leucócitos (4000 a 12000/mm ^3^ )	13.920 (10.723 -20.398)	15.150 (12.230-18.110)	0,97 (0,88 – 1,09)	0,681
Proteína C reativa (< 5,0 mg/L)	4,29 (1,54-13,59)	5,0 (1,65-58,19)	0,93 (0,83 – 1,05)	0,255
**Período 48h pós-operatório**				
Hb (11,0 a 14,5 g/dL)	10,6 (8,4-12,0)	10,6 (9,4-11,6)	1,02 (0,92 - 1,13)	0,688
Leucócitos (4000 a 12000/mm ^3^ )	12.980 (11.280-17.980)	13.500 (10.740-18.020)	1,01 (0,91 - 1,12)	0,835
Proteína C reativa (< 5,0 mg/L)	75,90 (49,55-118,19)	93,90 (64,89-151,12)	0,87 (0,77 - 0,99)	0,032
Reoperação na mesma hospitalização (%)	17 (26%)	7 (5,7%)	5,75 (2,24 - 14,74)	<0,001
Profilaxia adequada	58 (88%)	105 (85%)	0,27 (0,05 - 1,55)	0,144

Dados apresentados como medianas e percentis 25-75, número de casos ou porcentagem. IC: intervalo de confiança; CEC: circulação extracorpórea; ECMO: membrana de oxigenação extracorpórea; PS: pronto socorro; Hb: hemoglobina; UTI: unidade de terapia intensiva; OR: odds ratio; pré: período pré-operatório. *Magreza: índice antropométrico definido por valores de índice de massa corpórea ou relação peso/ estatura ≥ percentil 0,1 e < p 3 (≥Escore z -3 e <Escore Z-2). ^22^ **Eutrofia: índice antropométrico definido por valores de índice de massa corpórea ou relação peso/ estatura ≥ percentil 3 e ≤ p 85 (≥Escore z -2 e ≤ Escore Z+ 1). ^22^ ***Obesidade: índice antropométrico definido por valores de índice de massa corpórea ou relação peso/ estatura > percentil 97 e ≤ p 99,9 (≥Escore z +2 e ≤ Escore Z+ 3). ^22^ ****Prematuridade: idade gestacional ao nascimento < 37 semanas. ^22^

Fonte: Ribeiro. ^23^

### Recomendações quanto à profilaxia antimicrobiana

Utilizou-se cefuroxima por via venosa (dose=50 mg/kg) administrada na indução anestésica, repetida a cada 4 horas durante a cirurgia. Não é recomendada a administração de dose ao término da CEC. Após o término da cirurgia administra-se 30 mg/kg a cada seis horas até completar 24 horas de pós-operatório (quatro doses na UTI cirúrgica). Para crianças com mais de 30 kg, utiliza-se cefuroxima 1,5 g na indução anestésica e 750 mg a cada 4 horas durante a cirurgia e a cada 6 horas no pós-operatório por 24 horas. Para a análise da profilaxia antimicrobiana foram consultados os prontuários eletrônico e de papel.

### Análise estatística

A análise estatística foi realizada utilizando o programa estatístico SPSS versão 23.0 ( *SPSS Inc., Chicago, IL, EUA* ).

As variáveis numéricas foram expressas em mediana e intervalo interquartil (percentis 25 e 75). As variáveis categóricas foram apresentadas utilizando frequências absolutas e relativas. As diferenças entre dois grupos foram analisadas com o uso do teste de Mann-Whitney para variáveis numéricas após verificação de não normalidade pelo teste de Shapiro Wilk, e testes de Qui-quadrado ou teste de Fisher para as variáveis categóricas, quando adequado.

Para a análise dos fatores de risco para a infecção do sítio cirúrgico foi utilizado o modelo de regressão binária logística. Para este modelo as variáveis numéricas foram analisadas em decis. As variáveis de exposição com valor de p <0,1 na análise univariada foram escolhidas para a análise multivariada e o procedimento *forward LR* foi usado para a seleção de variáveis neste modelo final. Para cada possível preditor foi calculada a razão de chances, assim como seu intervalo de confiança de 95%. Significância estatística foi definida como valor de p <0,05.

## Resultados

Entre 1º de janeiro de 2011 e 31 de dezembro de 2018 foram operados 2378 pacientes. O número total final de casos foi de 66 pacientes. Nove casos foram pareados com apenas um controle, por inexistência de diagnóstico equivalente para ser pareado no período ou por outra infecção concomitante. O número final de controles após a randomização e adequação de critérios foi de 123 pacientes.

A incidência anual de infecção do sítio cirúrgico em pacientes submetidos a cirurgia cardíaca para cardiopatias congênitas no período de 2011 a 2018 na faixa etária de um a 19 anos variou de 2% a 3,8%.

Os diagnósticos de infecção de sítio cirúrgico foram: 29 casos (44%) de ISC superficial, 14 (21%) de ISC profunda e 23 (35%) como ISC órgão/ espaço, sendo 7 mediastinites, 5 osteomielites, 5 endocardites e 6 casos com dois diagnósticos associados, a saber osteomielite e mediastinite (3 casos); osteomielite e endocardite (2 casos) e 1 paciente com mediastinite associada à endocardite. Foram coletadas amostras de secreção do sítio cirúrgico de 50 pacientes (76%) e houve identificação de agente em 37 casos (74%): *Staphylococcus aureus* em 26 pacientes (70,3%), *Staphylococcus epidermidis* (6 pacientes - 16,2%), *Staphylococcus cohnii* (1 paciente - 2,7%), *Staphylococcus hominis* (1 paciente - 2,7%), *Enterococcus faecium* (1 paciente - 2,7%), *Acinetobacter haemolyticus* (1 paciente - 2,7%) *e Enterobacter cloacae complex* (1 paciente - 2,7%). Quanto ao perfil de sensibilidade, 29% dos estafilococos (10/34) foram resistentes à oxacilina e 71%,(24/34) sensíveis. *Enterococcus faecium* foi resistente a vancomicina. Em 8 pacientes (21%) houve também hemocultura positiva. Os agentes etiológicos identificados nas hemoculturas foram: *Staphylococcus aureus* (quatro ISC profundas e duas mediastinites), *Staphylococcus epidermidis* (uma osteomielite) e *Enterococcus faecium* (uma mediastinite).

Houve 6 óbitos (3,2%), apenas nos pacientes infectados e com diagnóstico de ISC órgão/espaço e hemocultura positiva em todos. Os agentes etiológicos identificados nas hemoculturas foram: *S aureus, S. epidermidis* e *E. faecium* .

Na avaliação da antibioticoprofilaxia, faltaram dados em 6% (04/66) no grupo caso e 13% (16/123) no grupo controle. Houve conformidade da profilaxia antimicrobiana com o protocolo institucional acima de 90%, sem diferença estatisticamente significante entre casos e controles (p=0,144).

**Figura 1:**
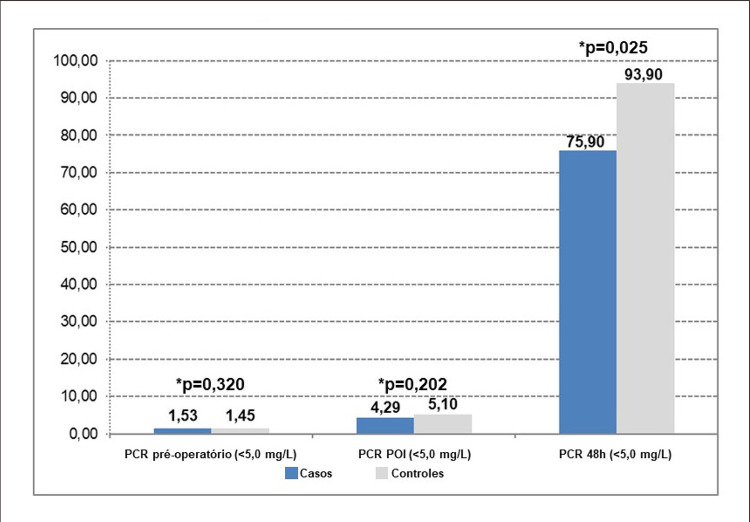
– Evolução dos valores em medianas da PCR nos pacientes de um a 19 anos submetidos à cirurgia cardíaca, 2011-2018

Os potenciais fatores de risco para ISC estão descritos na Tabela 1 e os fatores significantes obtidos na análise univariada estão descritos na Tabela 2 . Os lactentes, portadores de síndrome genética, pacientes pertencentes às categorias 3 e 4 do RACHS-1, antecedente de cirurgia realizada em anos anteriores e reoperação na mesma internação estavam sob maior risco para o desenvolvimento da infecção do sítio cirúrgico. Por outro lado, os pacientes com valores maiores da PCR após 48 horas de pós-operatório apresentaram menor risco para esta infecção ( Tabela 2 ). A Figura 1 ilustra a evolução dos valores da PCR nos pacientes de 1 a 19 anos submetidos à cirurgia cardíaca nos períodos pré, intra e pós–operatórios.

**Tabela 2:** – Fatores de risco significantes para infecção do sítio cirúrgico nos pacientes com um a 19 anos de idade submetidos à cirurgia cardíaca de 2011 a 2018: análise univariada

Variável de exposição	Grupo Caso (N=66)	Grupo Controle (N=123)	Razão de chances – OR (IC 95%)	p
Faixa etária lactente, N (%)	31 (47%)	36 (29,3%)	2,14 (1,15 - 3,98)	0,016
Síndrome genética, N (%)	13 (20%)	7 (5,7%)	4,03 (1,53 - 10,77)	0,005
RACHS-1 ≥3, N (%)	43 (65%)	44 (35%)	3,35 (1,79 - 6,28)	<0,001
Cirurgia prévia, N (%)	33 (50%)	43 (35%)	1,86 (1,01 - 3,41)	0,046
Proteína C reativa 48 hs PO (< 5,0 mg/L)	75,90 (49,55 – 118,19)	93,90 (64,89 – 151,12)	0,87 (0,77 - 0,99)	0,032
Reoperação na mesma hospitalização (%)	17 (26%)	7 (5,7%)	5,75 (2,24 - 14,74)	<0,001

Dados apresentados como medianas e percentis 25-75, número de casos ou porcentagem. IC: intervalo de confiança; h: horas; OR: odds ratio; PO: período pós-operatório; RACHS-1: Risk Adjustment in Congenital Heart Surgery, version 1.

Fonte: Ribeiro. ^23^

Os fatores de risco para a infecção do sítio cirúrgico obtidos na análise multivariada estão descritos na Tabela 3 .

**Tabela 3:** – Fatores de risco associados a infecção do sítio cirúrgico na análise multivariada nos pacientes com um a 19 anos de idade submetidos à cirurgia cardíaca de 2011 a 2018

Variável de exposição	OR	IC 95%	p
Faixa etária lactente	3,19	1,26 – 8,66	0,014
Síndrome genética	6,20	1,70 – 21,65	0,004
RACHS ≥3	8,40	3,30 – 21,34	<0,001
Proteína C reativa 48 hs PO	0,85	0,73 – 0,98	0,023

IC: intervalo de confiança; h: horas; OR: odds ratio; PO: período pós-operatório; RACHS-1: Risk Adjustment in Congenital Heart Surgery, version 1.

Fonte: Ribeiro. ^23^

A Figura central ilustra incidência da ISC e os fatores de risco encontrados no estudo.

## Discussão

A identificação da faixa etária de lactentes como fator de risco para infecção do sítio cirúrgico é biologicamente plausível visto que o processo de desenvolvimento do sistema imune na criança inicia-se na vida fetal e continua até a adolescência. O recém-nascido e o lactente possuem menor capacidade de resposta aos antígenos em comparação com as crianças mais velhas, adolescentes e adultos. Este achado corrobora para o papel preditor da baixa idade para ISC já descrito na literatura. ^1 , 3 , 4 , 6 , 14^

Neste estudo verificou-se que a presença de síndrome genética foi fator de risco para ISC. Estudos prévios como o de Costello et al., ^3^ Sen et al. ^4^ e Hatachi et al. ^15^ mostraram presença de alterações cromossômicas como preditoras para infecção do sítio cirúrgico em pós-operatório de cirurgia cardíaca para cardiopatia congênita. A síndrome de Down é a síndrome genética reconhecidamente mais associada a alterações imunes nos compartimentos celular, humoral e fagocítico e foi a mais prevalente em nossa população. A capacidade lítica dos polimorfonucleares é efetuada pela atividade de superóxidos e outros radicais, os quais provocam danos celulares oxidativos, eliminando fungos e bactérias como *Candida spp* . e *Staphyloccocus spp* . A enzima cobre-zinco-superoxidodismutase-1 (Cu-Zn-SOD-1) a qual converte superóxidos em peróxido de hidrogênio é codificada pelo gene *SOD1,* localizado no cromossomo 21. A carga genética extra determinada pela trissomia está relacionada a níveis elevados de Cu-Zn-SOD-1, o que reduz a quantidade de superóxidos em polimorfonucleares de portadores da trissomia do cromossomo 21. ^16 , 17^

Costello et al. ^3^ e Sen et al. ^4^ descreveram a associação entre a classificação da complexidade de procedimentos cirúrgicos para cardiopatias congênitas pelo RACHS-1 e o risco para ISC. Em nosso estudo identificou-se a categoria de RACHS-1 acima de 3 como fator de risco independente para ISC. Os procedimentos cirúrgicos relacionados às anatomias mais complexas podem acarretar maior tempo em sala cirúrgica, manejo mais intenso e prolongado dos tecidos com maior chance de contaminação do sítio cirúrgico e de dano celular. Neste estudo os tempos de cirurgia e de circulação extracorpórea não foram evidenciados como preditores de infecção. Outra consideração a ser feita é o fato de que o paciente com cardiopatia mais complexa pode ter uma condição clínica basal com potencial de descompensação hemodinâmica maior em relação a um paciente com alterações anatômicas mais simples. A alteração do débito cardíaco pode acarretar a redução da vascularização tecidual e colaborar para a infecção.

Quando analisamos a presença de cirurgia prévia realizada em anos anteriores, foi observado que estes pacientes estavam sob maior risco para ISC p=0,046 (IC 95%,1,01-3,41), OR=1,86. O histórico de cirurgia prévia é sugerido em publicações como fator de risco para ISC, ^14^ entretanto esta variável de exposição não permaneceu como fator de risco para a infecção do sítio cirúrgico quando foi submetida posteriormente à análise multivariada.

Os valores da proteína C reativa após 48 horas da operação significantemente superiores no grupo de pacientes não infectados foi um achado inesperado. Na análise multivariada, mostrou-se como um fator de proteção, p= 0,023 (IC 95%, 0,73-0,98), OR=0,85. Para cada incremento de um decil houve redução do risco de infecção do sítio cirúrgico em 15%.

A PCR é denominada proteína de fase aguda inflamatória. A inflamação é a resposta humoral e celular protetora do corpo humano à injúria. Ela engloba a ativação de diferentes cascatas como a do sistema complemento, das citocinas e da coagulação. No contexto da cirurgia cardíaca ela já é desencadeada na anestesia e aumentada pela incisão cirúrgica da pele e esternotomia e, finalmente é amplificada de forma robusta pela circulação extracorpórea (CEC). ^18^

No período de pós-operatório de cirurgia cardíaca os níveis séricos da PCR necessitam ser interpretados com cautela e avaliados em conjunto com dados clínicos, epidemiológicos, demais exames complementares e outros biomarcadores, pois níveis séricos elevados podem ser interpretados como uma complicação infecciosa e justificar a introdução de terapia antimicrobiana empírica ou prolongamento da profilaxia antimicrobiana. A antibioticoprofilaxia nos pacientes com PCR elevada não foi prolongada em função destes valores, e todos os pacientes do grupo controle que receberam algum antimicrobiano por suspeita de diagnóstico infeccioso no pós-operatório foram excluídos do estudo.

A evolução dos níveis séricos da PCR ilustrada na Figura 1 está em conformidade com dados da literatura a respeito do pico da PCR no segundo dia de pós-operatório. Jaworski et al. ^19^ realizaram estudo para avaliação da cinética da proteína C reativa em crianças com cardiopatia congênita submetidas à cirurgia cardíaca com CEC e observaram que os maiores níveis da PCR ocorreram no segundo dia de pós-operatório e que os valores eram altos mesmo na ausência de complicações infecciosas. ^19^ Tradicionalmente utilizada como marcador de infecção e de eventos cardiovasculares, a proteína C reativa atualmente está sendo apontada por novas evidências como uma proteína com papel ativo e relevante nos processos de inflamação e resposta do hospedeiro a infecções incluindo a via do sistema complemento, apoptose, fagocitose, liberação de óxido nítrico e produção de citocinas, particularmente a interleucina 6 e o fator de necrose tumoral alfa. Na presença de cálcio a PCR liga-se a polissacarídeos nos microrganismos e ativa a via clássica do complemento que promove a opsonização de patógenos. Há relatos de que a PCR pode mediar a resposta do hospedeiro ao *Staphylococcus aureus* promovendo o aumento da fagocitose bacteriana. Sproston et al. ^20^ mostraram a ação da PCR sobre a parede polissacarídica bacteriana. ^20^ Ponderando-se que *Staphylococcus aureus* é o principal microrganismo identificado nas infecções do sítio cirúrgico na maioria dos estudos, o que também foi constatado em nossos casos, o valor mais elevado da PCR nas 48 h pós-operatórias nos pacientes do grupo controle demonstra a possibilidade de a mesma exercer um papel de opsonina, fator protetor para a ISC. Os valores menores da PCR nos pacientes infectados do grupo caso em relação aos do grupo controle enfatizam a importância de não interpretarmos esta proteína de forma isolada e apenas como marcador de infecção.

D’Souza et al. ^21^ realizaram estudo observacional prospectivo do valor preditivo de biomarcadores como a PCR, procalcitonina, lactato, neutrófilos e linfócitos, plaquetas para o diagnóstico de infecção bacteriana após cirurgia cardíaca na população pediátrica. Foram incluídos 368 pacientes e foi descrito como sendo o maior estudo com foco neste assunto publicado até o momento. Apesar disto, eles concluíram que a diferenciação entre infecção e estado inflamatório pós-operatório permanece difícil nesta faixa etária. As medidas longitudinais da PCR e procalcitonina e monitoramento das alterações clínicas que ocorrem na evolução do paciente no período perioperatório são informações valiosas e devem ser consideradas na decisão sobre o uso racional de antimicrobianos no pós-operatório. ^21^

A utilização de biomarcadores como PCR e procalcitonina no pós-operatório de cirurgia cardíaca infantil requer uma análise detalhada e atrelada a uma situação clínica para evitar diagnósticos equivocados de infecções, prescrição indiscriminada de antimicrobianos e seleção de microrganismos multirresistentes. São necessários estudos prospectivos multicêntricos para confirmação e consolidação dos achados.

O presente estudo apresenta limitações relacionadas ao número total de pacientes infectados e ao desenho retrospectivo unicêntrico. O fato da infecção do sítio cirúrgico ser um desfecho raro pode prejudicar a análise de fatores preditores. A análise retrospectiva de dados em prontuários eletrônicos e físicos apresenta dificuldades quanto à acurácia das informações. A população de um centro único de referência pode ter características peculiares. A realização de estudos multicêntricos e a ampliação das linhas de investigação poderão validar os achados deste estudo.

## Conclusão

Neste estudo, foram identificados fatores de risco para infecção do sítio cirúrgico que não são modificáveis antes da cirurgia (idade, doença genética, por exemplo). Deste modo, a prevenção desta infecção requer o cumprimento rigoroso das medidas de prevenção de infecção, tais como reduzir tempo de internação pré-operatório, utilizar antibioticoprofilaxia individualizada, manipulação cuidadosa de sondas, cateteres e curativos no pós-operatório.

Outro ponto a destacar foi o valor mais elevado da PCR nas primeiras 48 hs após a cirurgia ter sido demonstrado como fator protetor para a ocorrência da ISC. O provável papel imunomodulador da proteína C reativa no período pós-operatório da cirurgia cardíaca requer maior investigação, evitando que seu resultado seja interpretado exclusivamente como marcador de infecção e levando ao uso inapropriado de antimicrobianos.
