# Supplementary material for: Fatores de Risco para Infecção da Ferida Operatória em Pacientes Submetidos à Cirurgia Cardíaca Pediátrica
Source: Arq Bras Cardiol. 2023 Dec 7;120(12):e20220592. [Article in Portuguese] doi: 10.36660/abc.20220592 (PMC10789367; doi:10.36660/abc.20220592)
Supplement: Translation

# Translation: Risk Factors for Surgical Site Infection in Patients Undergoing Pediatric Cardiac Surgery

**Authors:** Anna Christina de Lima Ribeiro, Rinaldo Focaccia Siciliano, Antonio Augusto Lopes, Tania Mara Varejão Strabelli

**Affiliations:** 1 Brazil Instituto do Coração do Hospital das Clínicas da Faculdade de Medicina da Universidade de São Paulo - Cardiologia Pediátrica e Cardiopatias Congênitas do Adulto, São Paulo, SP – Brazil; 2 Brazil Equipe de Controle de Infecção - Instituto do Coração do Hospital das Clínicas da Faculdade de Medicina da Universidade de São Paulo, São Paulo, SP – Brazil

**Keywords:** Risk Factors, Congenital Heart Defects, Postoperative Complications, Cardiac Surgical Procedures, Surgical Wound Infection

## Abstract

**Background:**

Surgical site infection is an important complication after pediatric cardiac surgery, associated with increased morbidity and mortality.

**Objectives:**

We sought to identify risk factors for surgical site infection after pediatric cardiac surgeries.

**Methods:**

A case-control study included patients aged between 1 year and 19 years and 11 months of age, submitted to cardiac surgery performed at a tertiary cardiac center from January 1 ^st^ , 2011, through December 31, 2018. Charts were reviewed for pre-, intra, and postoperative variables. We identified two randomly selected control patients with the same pathophysiological diagnosis and underwent surgery within thirty days of each index case. Univariate and multivariate logistic regression analyses were performed to identify risk factors. Statistical significance was defined as p<0.05.

**Results:**

Sixty-six cases and 123 controls were included. Surgical site infection incidence ranged from 2% to 3.8%. The following risk factors were identified: Infant age (OR 3.19, 95% CI 1.26 to 8.66, p=0.014), presence of genetic syndrome (OR 6.20, CI 95% 1.70 to 21.65, p=0.004), categories 3 and 4 of RACHS-1 (OR 8.40, CI 95% 3.30 to 21.34, p<0.001), 48 h C-reactive protein level range was detected as a protective factor for this infection (OR 0.85, 95% CI 0.73 to 0.98, p=0.023).

**Conclusions:**

The risk factors defined in this study could not be modified. Therefore, additional surveillance and new preventive strategies need to be implemented to reduce the incidence of surgical site infection. The increased CRP in the postoperative period was a protective factor that needs further understanding.

## Introduction

Congenital heart disease is a growing public health concern worldwide, especially in developing countries. Despite the improvement in pediatric cardiac surgery, the need for specialized services and the limitations of human beings and financial resources are challenging for those countries. ^1 , 2^ Surgical site infection (SSI) is a relevant complication associated with increased morbidity and mortality and higher costs to the health system. ^1 - 6^ In pediatric cardiac surgical patients, reported SSI incidence rates vary from 0.2% to 4.8%. ^7^

Only a few studies have focused on identifying risk factors for SSI after cardiac surgery in children older than one-year-old, as the focus is on the neonatal period. There are no studies with this specific focus on the pediatric population in Brazil. This study aims to contribute to expanding knowledge on the subject.

Prior studies have identified age under one month, genetic syndrome, high American Society of Anesthesiology (ASA) score, cyanogenic heart disease, intraoperative hypothermia, preoperative hospitalization for more than 48 hours, duration of surgery, and cardiopulmonary bypass (CPB) time, use of multiple procedures during surgery, number of red blood cell transfusions and delay in completion of sternal closure as risk factors for SSI. ^1 , 3 , 8^

The study’s primary objective was to identify risk factors for infection at the surgical site after cardiac surgery for congenital heart disease with and without cardiopulmonary bypass (CPB) in children. As a secondary endpoint, the incidence and microbiology of infections were evaluated.

## Methods

The Scientific Committee and the Ethics Committee for Analysis of Research Projects of the tertiary cardiology university hospital approved this study. The free and informed consent form was waived.

### Patients

A retrospective 1:2 case-control study design was used to identify risk factors. This study included 189 patients aged between 1 and 19 years old who had undergone cardiac surgery at a tertiary cardiac center specialized in high-complexity pediatric care cardiovascular surgery from January 1 ^st^ , 2011, to December 31, 2018. According to the World Health Organization (WHO) definition, the age group of adolescents in pediatrics encompasses 10 to 19 years. ^9^ We adopted this standardization.

### Inclusion criteria

Case definition: Patients with congenital heart disease aged between one and 19 years old who underwent cardiac surgery with surgical site infection (SSI).

Control definition: Patients with congenital heart disease aged between one and 19 years old who underwent cardiac surgery without SSI.

### Exclusion criteria

Newborn patients and infants until the first year of life (29 days of age until 11 months and 29 days).

Patients undergoing cardiac surgery for diagnoses other than congenital heart disease, such as cardiomyopathies, pericardiopathies, cardiac tumors, chronic rheumatic disease, patients indicated for heart transplantation, patients indicated for placement of an electronic device or circulatory assistance device in the absence of congenital heart disease.

### Selection of cases and controls

All SSI diagnoses were confirmed by the Hospital Infection Control Unit (UCIH) team in line with defining diagnostic criteria according to Centers for Disease Control and Prevention (CDC), Atlanta, USA. ^10 - 12^

For each case, two controls were selected and combined by diagnosis of heart disease and by surgery date (±30 days) to minimize pre and/or intraoperative differences. The controls were randomly selected using the Excel program. Congenital heart diseases were divided into categories according to the pathogenic, pathophysiological basis and arterial oxygen saturation in four groups: group 1) acyanotic congenital heart disease with an obstructive lesion, group 2) acyanotic congenital heart disease with left to - right shunt defect, group 3) cyanotic congenital heart disease with obstruction to pulmonary blood flow and group 4) cyanotic congenital heart disease with increased pulmonary blood flow. ^13^ Pre, intra, and postoperative data (demography, clinical, laboratory values) were recorded, and exposure variables were analyzed if considered biologically relevant and according to the literature review. ( Table 1 ) Considering that the transfusion of blood products can be an important risk factor, having received at least one unit of any blood product was considered a potential risk factor.

**Table 1:** Potential risk factors for surgical site infection by univariate analysis in patients with congenital heart disease aged between one and 19 years old, who underwent cardiac surgery, 2011-2018

Exposure	Case Group (N=66)	Control Group (N=123)	OR (CI 95%)	p
**Preoperative data Age range, N (%)**				
Infant (1-2 years old)	31 (47%)	36 (29.3%)	2.14 (1.15 - 3.98)	0.016
Children (3-9 years old)	14 (21.2%)	39 (31.7%)	0.58 (0.28 - 1.17)	0.128
Teenager (10-19 years old)	21 (31.8%)	48 (39%)	0.73 (0.39 -1.37)	0.327
Underweight, N (%)*	16 (24.3%)	25 (20%)	1.25 0.61- 2.56)	0.534
Normal weigth, N (%)**	48 (72.7%)	86 (70%)	1.10 (0.56 - 2.15)	0.772
Obesity, N (%)***	2 (3%)	12 (9.8%)	0.29 (0.63 - 1.33)	0.111
Prematurity, N (%)****	3 (4.5%)	3 (2.5%)	1.90 (0.37 - 9.71)	0.438
Genetic syndrome, N (%)	13 (20%)	7 (5.7%)	4.03 (1.53 - 10.77)	0.005
Saturation O _2_ (%)	96 (80-98)	96 (90-98)	0.91 (0.65 - 1.25)	0.570
RACHS-1 ≥3, N (%)	43 (65%)	44 (35%)	3.35 (1.79 - 6.28)	<0.001
Preoperative procedures, N (%)	5 (7.5%)	5 (4%)	1.83 (0.53 - 6.94)	0.311
Previous surgery, N (%)	33 (50%)	43 (35%)	1.86 (1.01 - 3.41)	0.046
Total days of preoperative hospitalization, N (%)	3 (1.75-8)	3 (1-5)	1.09 (0.96 - 1.24)	0.188
**Preoperative hospitalization**				
ICU, N %	5 (7.5%)	5 (4.9%)	1.70 (0.59 - 4.93)	0.324
ER, N %	7 (10%)	8 (6.5%)	1.59 (0.47 - 5.45)	0.454
Nursery, N (%)	57 (86%)	115 (93%)	0.44 (0.16 - 1.20)	0.110
**Preoperative Laboratory values**				
Hb (11.0 to 14.5 g/dL)	13.7 (12.6-16.0)	13.8 (12.8-15.3)	1.01 (0.91 - 1.24)	0.870
**Intraoperative data**				
Duration of surgery (minutes)	295 (225-361)	293(230-350)	1.00 (0.90 - 1.11)	0.997
CPB (minutes)	98 (59-127)	98 (59-127.7)	0.99 (0.89 - 1.12)	0.992
Aortic Cross-Clamp (minutes)	65 (36-87)	63 (40-99)	0.975 (0.87 - 1.09)	0.669
Hypothermia	57 (86%)	112 (91%)	0.622 (0.24 - 1.58)	0.321
Any transfusion, N (%)	36 (54.5%)	65 (53%)	1.07 (0.80 - 1.95)	0.823
Red blood cell transfusion, N (%)	34 (51%)	54 (44%)	1.35 (0.74 - 2.47)	0.318
ECMO, N (%)	2 (3%)	2 (1.6%)	1.89 (0.26 - 13.73)	0.529
Delayed sternal closure, N (%)	3 (4.5%)	2 (1.6%)	2.88 (0.46 -17.69)	0.253
Lowest glucose value (mg/dL)	95 (85-110)	96 (84-109)	1.02 (0.92 - 1.14)	0.604
Highest glucose value (mg/dL)	169 (131-191)	164 (131-195)	1.02 (0.92 - 1.13)	0.743
**Postoperative data - Laboratory values**				
**Immediate postoperative**				
WBC count (4.000 to 12.000/mm ^3^ )	13.920 (10.723 -20.398)	15.150 (12.230-18.110)	0.97 (0.88 - 1.09)	0.681
C reactive protein (< 5.0 mg/L)	4.29 (1.54-13.59)	5.0 (1.65-58.19)	0.93 (0.83 - 1.05)	0.255
**48 h postoperative**				
Hb (11.0 to 14.5 g/dL)	10.6 (8.4-12.0)	10.6 (9.4-11.6)	1.02 (0.92 - 1.13)	0.688
WBC count (4.000 to 12.000/mm ^3^ )	12.980 (11.280-17.980)	13.500 (10.740-18.020)	1.01 (0.91 - 1.12)	0.835
C-Reactive Protein (< 5.0 mg/L)	75.90 (49.55-118.19)	93.90 (64.89-151.12)	0.87 (0.77 - 0.99)	0.032
Reoperation in the same hospitalization (%)	17 (26%)	7 (5.7%)	5.75 (2.24 - 14.74)	<0.001
Appropriate antibiotic prophylaxis	58 (88%)	105 (85%)	0.27 (0.05 - 1.55)	0.144

Data are presented as median with interquartile range (25-75 ^th^ ) and numbers and percentiles. CI: confidence interval; CPB: cardiopulmonary bypass; ECMO: extracorporeal membrane oxygenation; ER: emergency room; Hb: hemoglobin; ICU: intensive care unit; O _2_: oxygen; OR: odds ratio; Pre: preoperative period; WBC: white blood cell. * Underweight: anthropometric index defined by values of body mass index or weight/height ratio ≥ 0.1 percentile and < p 3 (≥ z-score -3 and < z-score-2). ^22^ ** Normal weight: defined anthropometric index by body mass index values or weight-to-height ratio ≥ 3 ^rd^ percentile and ≤ p 85 (≥ z-score -2 and ≤ z-score 1). ^22^ *** Obesity: anthropometric index defined by body mass index values or ratio weight/height > 97 ^th^ percentile and e ≤ p 99.9 (≥ z-score +2 and ≤ z-score + 3). ^22^ **** Prematurity: gestational age at birth < 37 weeks. ^22^

Source: RIBEIRO, ^23^

### Antibiotic prophylaxis recommendations

Intravenous cefuroxime (dose=50mg/kg) was administered during anesthetic induction and repeated every 4 hours during surgery. Dose administration at the end of CPB is not recommended. After the end of the surgery, 30mg/kg is administered every six hours until completed 24 hours postoperatively (four doses in the surgical ICU). For children weighing more than 30 kg, cefuroxime 1.5g is used during anesthetic induction and 750mg every 4 hours during surgery and every 6 hours postoperatively for 24 hours. For the analysis of antimicrobial prophylaxis, electronic and paper records were consulted.

### Statistical analysis

SPSS software version 23.0 (SPSS Inc., Chicago, IL, USA) was used for statistical analysis. Numerical variables were expressed as median and interquartile range (25 ^th^ and 75 ^th^ percentile). Categorical variables were presented using absolute and relative frequencies. Differences between the two groups were analyzed using the Mann-Whitney test for numeric variables after checking for non-normality using the Shapiro-Wilk test and Chi-square test or Fisher’s test for categorical variables, when appropriate. A binary logistic regression model was used to identify risk factors for SSI. In regression analysis, numerical variables were categorized into deciles. Exposure variables with a probability value below 0.1 were considered in the univariate analysis for inclusion in a multivariate model. Procedure *LR Forward* was used to select each variable in the final model. The odds ratio (OR) ratio with 95% confidence intervals (CI) was calculated. Statistical significance was defined as p<0.05.

## Results

Between January 1 ^st^ , 2011, and December 31, 2018, 2378 patients underwent cardiac surgery, of whom 66 had SSI. These cases were matched with two controls by date of operation and congenital heart defect group. Nine cases were matched with only one control due to a lack of equivalent diagnosis to be matched during the period or due to another concomitant infection. The final number of controls after randomization and matching criteria was 123 patients.

The annual incidence of SSI after cardiac surgery for congenital heart disease in children over the eight-year study ranged from 2.0% to 3.8%.

The type of SSI was superficial incisional in 29 patients (44%), deep incisional in 14 (21%), and organ space in 23 (35%), with seven mediastinitis, five osteomyelitis, five endocarditis, and six cases with two associated diagnoses, namely osteomyelitis and mediastinitis (three cases); osteomyelitis and endocarditis (two cases) and one patient with mediastinitis associated with endocarditis. Surgical site cultures were obtained in 50 patients (76%) and were positive in 37 cases (74%): *Staphylococcus aureus* was identified in 26 patients (70.3%), *Staphylococcus epidermidis* in six (16.2%), *Staphylococcus cohnii* in one (2.7%), *Staphylococcus hominis* in one (2.7%), *Enterococcus faecium* in one (2.7%), *Acinetobacter sp* in one (2.7%) and *Enterobacter cloacae* complex in one (2.7%). As to the sensitivity profile, 29% of the staphylococci (10/34) were resistant to oxacillin and 71% (24/34) sensitive. Enterococcus faecium was resistant to vancomycin. Blood cultures were positive in eight patients (21%) out of 37 cases. The etiological agents identified in blood cultures were *Staphylococcus aureus* (four deep SSIs and two mediastinitis), *Staphylococcus epidermidis* (one osteomyelitis), and *Enterococcus faecium* (one mediastinitis).

Six patients died (3.2%). All had SSI organ space and positive blood culture. Etiological agents identified in blood cultures were *Staphylococcus aureus, Staphylococcus epidermidis, and Enterococcus faecium* .

Regarding antibiotic prophylaxis, the percentage of missing data was around 6% (four out of 66) in the case group and 13% (16 out of 123) in the control group. There was compliance of antimicrobial prophylaxis with the institutional protocol above 90%, with no statistically significant difference between cases and controls (p=0.144).

The potential risk factors for SSI are listed in Table 1 . Those that are significant by univariate analysis are shown in Table 2 . Infant patients, those with genetic syndrome, RACHS-1 categories 3 and 4, and those who required surgery performed in previous years and reoperation in the same hospitalization had a higher risk of developing SSI. Conversely, patients with higher C-reactive protein (C-RP) levels in the 48 postoperative hours presented a lower risk for SSI ( Table 2 ). The evolution of the serum levels of C-RP in the preoperative and postoperative periods is illustrated in Figure 1 . Independent risk factors for SSI in multivariate analysis are displayed in Table 3 . The annual incidence of SSI and risk factors for SSI are illustrated in the Central Figure.

**Table 2:** – Significant risk factors for surgical site infection by univariate analysis in patients with congenital heart disease aged between one and 19 years old, who underwent cardiac surgery, 2011-2018: univariate analysis

Exposures	Case Group (N=66)	Control Group (N=123)	OR (CI 95%)	p
Infant age, N (%)	31 (47%)	36 (29.3%)	2.14 (1.15 - 3.98)	0.016
Genetic syndrome, N (%)	13 (20%)	7 (5.7%)	4.03 (1.53 - 10.77)	0.005
RACHS-1 ≥3, N (%)	43 (65%)	44 (35%)	3.35 (1.79 - 6.28)	<0.001
Previous surgery, N (%)	33 (50%)	43 (35%)	1.86 (1.01 - 3.41)	0.046
C-reactive protein 48 h PO (<5.0 mg/L)	75.90 (49.55 - 118.19)	93.90 (64.89 - 151.12)	0.87 (0.77 - 0.99)	0.032
Reoperation in the same hospitalization, N (%)	17 (26%)	7 (5.7%)	5.75 (2.24 - 14.74)	<0.001

Data are presented as median with interquartile range (25-75 ^th^ ) and numbers and percentiles. CI: confidence interval; h: hours; OR: odds ratio; PO: postoperative period; RACHS-1: Risk Adjustment in Congenital Heart Surgery, version 1.

Source: RIBEIRO. ^23^

**Figure 1:** – C-reactive protein level range (median) evolution in patients with congenital heart disease aged between one year and 19 years old, who underwent cardiac surgery, 2011-2018

**Table 3:** – Risk factors for surgical site infection by multivariate analysis in patients with congenital heart disease aged between one and 19 years old, submitted to cardiac surgery, 2011-2018

Variables	OR	CI 95%	p
Infant age	3.19	1.26 – 8.66	0.014
Genetic syndrome	6.20	1.70 – 21.65	0.004
RACHS ≥3	8.40	3.30 – 21.34	<0.001
C-reactive protein 48h PO	0.85	0.73 – 0.98	0.023

CI: confidence interval; h: hours; OR: odds ratio; PO: postoperative period; RACHS-1: Risk Adjustment in Congenital Heart Surgery, version 1

Source: RIBEIRO ACL, 2022

## Discussion

The infant age group was identified as a risk factor for SSI. Previous studies have already described the predictive role of young age for SSI. The process of immune system development in children begins in fetal life and continues through adolescence. The newborn and infant are less able to respond to antigens than older children, adolescents, and adults. ^1 , 3 , 4 , 6 , 14^

Genetic syndrome was a predictor for SSI in our study. Costello et al., ^3^ Sen et al. ^4^ and Hatachi et al. ^15^ observed chromosomal changes as predictors of SSI in the postoperative period of cardiac surgery for congenital heart disease. Down syndrome is the most common recognizable genetic syndrome associated with immunological changes in the cellular, humoral, and phagocytic compartments and is the most prevalent in our population. The lytic capacity of neutrophils polymorphonuclears is affected by the activity of superoxides and other radicals, which cause oxidative cellular damage, eliminating fungi and bacteria such as *Candida spp* . and *Staphylococcus spp.* The copper-zinc-superoxydodismutase-1 enzyme (Cu-Zn-SOD-1) that converts superoxides into hydrogen peroxide is encoded by the SOD1 gene, located on chromosome 21. The extra genetic load determined by trisomy is related to high levels of Cu-Zn-SOD-1, which reduces the amount of superoxides in polymorphonuclear patients with chromosome 21 trisomy. ^16 , 17^

RACHS-1 categories 3 to 4 patients were found to have a 3 times higher risk for SSI. The association between the complexity of surgical procedure evidenced by RACHS-1 and the risk for SSI had already been described by Costello et al. ^3^ and Sen et al. ^4^ Complex and prolonged cardiovascular operations may increase cardiac surgery time, tissue management, and cellular damage. Patients with complex congenital heart defects might have a baseline clinical condition prone to hemodynamic decompensation. Changes in cardiac output may reduce tissue vascularization and contribute to infection. However, the surgery and cardiopulmonary bypass duration were not statistically significantly associated with SSI in our study.

When we analyzed the influence of previous surgery performed in recent years, it was associated with SSI p=0.046 (CI 95%,1.01-3.41), OR=1.86. The previous cardiac surgery is suggested in publications as a risk factor for SSI; ^14^ however, this exposure variable did not remain a risk factor for infection in the surgical site after multivariate analysis by logistic regression.

The fact that the levels of C-reactive protein after 48 hours of operation were significantly higher in the group of non-infected patients was an unexpected finding, p=0.032 (IC 95%, 0.77-0.99), OR=0.87. In multivariate analysis, it was shown to be a protective factor, p=0.023 (95% CI, 0.73-0.98) OR=0.85. For each additional C-RP 48-hour decile, surgical site infection risk was reduced by 15%.

Inflammation is the human body’s protective humoral and cellular response to injury. It involves the activation of different cascades, such as the complement system, cytokines, and coagulation. In the context of cardiac surgery, it is already triggered in anesthesia, is increased by the surgical incision of the skin and sternotomy, and is robustly amplified by cardiopulmonary bypass (CPB). ^18^

In the postoperative period of cardiac surgery, the serum levels of C-RP need to be interpreted with caution and evaluated together with clinical, and epidemiological data, other complementary exams, and other biomarkers. High C-RP serum levels may be interpreted as an infectious complication and cause the introduction of empirical antimicrobial therapy or prolongation of antimicrobial prophylaxis. Antibiotic prophylaxis in patients with high CRP was not prolonged due to these values, and patients who received some antimicrobial drug due to suspected infectious diagnosis in the postoperative period were excluded from the control group.

The evolution of C-RP serum levels illustrated in Figure 1 follows the literature data on the C-RP peak on the second postoperative day. Jaworski et al. ^19^ conducted a study to evaluate the kinetics of C-reactive protein in children with congenital heart disease undergoing cardiac surgery with CPB. They observed that the highest levels of C-RP occurred on the second postoperative day and that the values were high even in the absence of infectious complications. ^19^ Traditionally used as a marker of infection and cardiovascular events, C-reactive protein is currently being pointed out by new evidence as a protein with an active and relevant role in the processes of inflammation and host response to infections, including the complement system pathway, apoptosis, phagocytosis, release of nitric oxide and production of cytokines, particularly interleukin 6 and tumor necrosis factor-alpha. In the presence of calcium, C-RP binds to polysaccharides in microorganisms and activates the classic complement pathway that promotes the opsonization of pathogens. There are reports that C-RP can mediate the host’s response to *Staphylococcus aureus* by promoting an increase in the phagocytosis of this bacterium. Sproston et al. ^20^ showed the action of C-RP on the bacterial polysaccharide wall. ^20^ Considering that *Staphylococcus aureus* is the most frequent main agent of surgical site infections, described in the literature and was also the most frequent agent in patients in the case group, the highest 48-hour C-RP level range in not infected patients demonstrates the possibility of performing a role of opsonin, a protective effect for SSI. It is of great importance that this protein should not be interpreted as only a marker of infection.

D’Souza et al. ^21^ carried out a prospective observational study of the predictive value of biomarkers such as C-RP, procalcitonin, lactate, neutrophils and lymphocytes, and platelets for the diagnosis of bacterial infection after cardiac surgery in the pediatric population. Three hundred sixty-eight patients were included, and it was described as the largest study focusing on this subject published until March 2022. Nevertheless, they concluded that this age group’s differentiation between infection and postoperative inflammatory status remains difficult. The longitudinal measurements of C-RP and procalcitonin and monitoring of clinical changes that occur in the evolution of the patient in the perioperative period are valuable information. They should be considered when deciding on the rational use of antimicrobials in the postoperative period. ^21^

Using biomarkers such as C-RP and procalcitonin in the postoperative period of child cardiac surgery requires a detailed analysis to avoid misdiagnosis of numerous infections, indiscriminate prescription of antimicrobials, and selection of multi-resistant microorganisms. Prospective multicenter studies are needed to confirm and consolidate the findings.

The present study has limitations. Surgical site infection is a rare outcome; thus, it can impair predictive factors analysis. The retrospective design of the study and the analysis of data in electronic and physical records presented difficulties related to the accuracy of the information. The population of a single referral center may have peculiar characteristics. Multicenter studies can validate the findings of this study.

## Conclusions

In conclusion, only non-modifiable risk factors for SSI were identified, such as patient age and presence of genetic syndrome. Therefore, infection prevention requires strict compliance with measures such as shortened preoperative hospital stay, individualized surgical prophylaxis, and careful handling of probes, catheters, and postoperative dressings.

Another point to highlight is that the higher value of CRP in the 48 hours after surgery in patients in the control group has been shown to be a protective factor for SSI. The likely immunomodulatory role of C-reactive protein in the postoperative period of cardiac surgery needs further investigation, preventing its result from being interpreted exclusively as a marker of infection, leading to inappropriate antimicrobials.
